# Prevention of *Salmonella* contamination of finished soybean meal used for animal feed by a Norwegian production plant despite frequent *Salmonella* contamination of raw soy beans, 1994–2012

**DOI:** 10.1186/s13028-014-0041-7

**Published:** 2014-07-11

**Authors:** Martin Wierup, Thor Kristoffersen

**Affiliations:** 1 Department of Biomedical Sciences and Veterinary Public Health, Swedish University of Agricultural Sciences, Uppsala, SE-75007, Sweden; 2 Denofa AS, Øraveien 2, N-1630 Gamle, Fredrikstad, Norway

**Keywords:** Salmonella, Soy bean, Crushing plant, Soybean meal, Animal feed control, Food safety, Pre harvest control, Animal feed material, Compound animal feed

## Abstract

**Background:**

*Salmonella* contaminated animal feed is a major source for introducing *Salmonella* into the animal derived food chain. Because soybeans frequently are contaminated with *Salmonell*a, soybean meal used as animal feed material, a by-product of a “crushing plant” which produces oil from soybeans, can be important source of *Salmonella* in the animal feed.

We report the successful control of *Salmonella* from 1994 to 2012 in a Norwegian crushing plant producing soybean meal from imported soy beans. The results are based on an officially supervised HACCP based program including annual testing of around 4000 samples.

**Results:**

During the 19-year period, 34% of samples collected during unloading of ships delivering soybeans yielded *Salmonella;* the proportion of samples from ships that yielded *Salmonella* varied from 12-62% each year. Dust samples from all shiploads from South America yielded *Salmonella*. In total 94 serovars of *Salmonella* were isolated, including nine (90%) of the EU 2012 top ten serovars isolated from clinical cases of salmonellosis in humans, including major animal pathogenic serovars like *Spp*. Typhimurium and Enteritidis.

The effectiveness of the HACCP based control was indicated by a low prevalence of *Salmonella* contamination in the clean area of the plant, which is considered to be the main reason for the successful prevention of *Salmonella* in the end product. Despite extensive testing, no sample from the finished soybean meal product was found to be *Salmonella* contaminated.

**Conclusions:**

This study shows that a HAACP-based control program in a soybean crushing plant can produce *Salmonella* free soybean meal despite frequent *Salmonella* contamination of raw soybeans. That approach is suggested as an effective way to minimize the risk of *Salmonella* exposure of the animal feed mills and contamination of the subsequent animal feed chain.

## Background

*Salmonella* contaminated animal feed is a major source for introducing *Salmonella* into the animal feed and food chain [1],[2]. A striking example emphasizing the potential of contaminated animal feed to act as a source of *Salmonella* infections in humans occurred when *S.* Agona emerged as a public health problem in several countries due to the spread of contaminated imported fish meal used as animal feed. In the USA a rapid increase of human infections with *S.* Agona occurred from 1968 to 1972 [3], and a similar increase of human infections with *S.* Agona occurred simultaneously in European countries. Since then, *S.* Agona has been among the most prevalent serotypes in humans. It is estimated that up to 2001 the serotype caused more than one million human illnesses in the USA alone since it was introduced in animal feed 1968 [1].

In the EU increasing focus has been directed to the prevention of *Salmonella* contamination during animal feed production [4],[5]. Major animal feed materials that often are reported to be *Salmonella* contaminated are meals and expellers (cakes) from the oil crushing industry, e.g. soybean meals, rapeseed, babassu, coconut expeller and palm kernel expeller [6]. The control and elimination of *Salmonella* contamination in the crushing plants would therefore be an important way to prevent *Salmonella* introduction into EU farms. Control of *Salmonella* in crushing plants is feasible if recontamination is prevented since the production process reaches temperatures that would eliminate *Salmonella*.

Crushing plants provide soybean meal and other meals to feed mills for the production of animal feeds. Data have been presented on the successful prevention of *Salmonella* contamination in feed mills in Scandinavian countries [7],[8], but the only report on prevention and control of *Salmonella* in a crushing plant was a rape seed plant [9]. Soybean meal is a much more common ingredient of animal feed than rape seed, and this report is the first from a soybean crushing plant.

## Methods

### Crushing plant

The company – Denofa AS – has its crushing plant situated in Fredrikstad, 100 km south of Oslo, Norway. During the period studied (1994–2012) the plant has increased its capacity from 375,000 tonnes to 420,000 tons of non-GMO soybeans per year, producing today around 330,000 tonnes of soybean meal products, 85,000 tonnes of crude soybean oil and 2,500 tonnes of soy lecithin.

### Supply chain for raw materials

The soy beans are imported mainly from Brazil. The production areas are in the interior of the country, the majority coming from the state of Mato Grosso. After harvest the beans are stored in large warehouses close to the farming areas. From there they are transported in trucks and barges about 2,500 km to the port terminals. About each month the Denofa plant receives handy size vessels (with 25–35,000 tonnes of soybeans) at Denofa’s own port close to the crushing plant.

### Markets

The soybean meal from Denofa is distributed to animal feed mills mainly in Norway, Sweden and Finland, for the production of compound animal feed mainly to cows, pigs and poultry. About 50% of the meal is delivered by sea in small coaster vessels; the rest is delivered to the clients by truck.

### Production process

**Figure 1:**
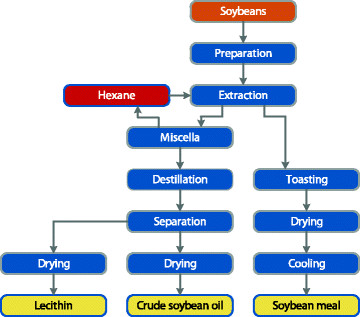
Flow sheet showing the production of soybean meal and associated products at the Denofa crushing plant.

The production is based on a traditional extraction process where the soybean oil is separated from the protein rich part by solvent extraction, as illustrated in Figure 1. After the oil has been extracted the meal is subject to toasting via direct steam treatment. Temperature reaches a minimum 105°C after a residence time of about 30 minutes. Processing conditions during toasting are considered to be of crucial importance for removal of eventual *Salmonella*. Afterwards, the toasting meal is dried, cooled and sent to dedicated storage areas.

### Control of Salmonella

#### Overall strategy

The plant produces soybean meal according to Number 2.18.3 in Part C of Commission Regulation (EC) No 68/2013 of 16 January 2013 on the Catalogue of animal feed materials. Supplying *Salmonella* free products is a vital part of the business strategy. In line with this strategy and legal requirements, the plant has a declared ambition to deliver only *Salmonella*-safe animal feed materials to customers/animal feed mills. This means that in addition to the HACCP programme described below, the product is not used in the production of animal feed until it has tested negative for *Salmonella* contamination. The plant is certified according to the standard “GMP + B2 (2010) Production of Animal feed Ingredients” [10].

#### Major preventive measures

In order to reduce the risk of *Salmonella* contamination to an absolute minimum, top priority is given to the following:

• The production site is carefully segregated into “unclean and clean zones”.

• Strict procedures and rules are established regarding: training of personnel, equipment design, operating conditions, buildings, logistics, maintenance and cleaning.

• Extensive sampling at critical control points, and appropriate action taken when *Salmonella* is isolated.

• Frequent inspections and audits.

#### Legislative demands

The requirements for hygienic animal feed production is laid down in the Norwegian regulation on animal feed hygiene (FOR 2010-01-14 nr 39: Forskrift om fôrhygiene) in which the corresponding EU legislation is adopted (Regulation (EC) No 183/2005 of the European Parliament and of the Council of 12 January 2005) laying out the requirements for animal feed hygiene. It is required that the animal feed manufacturer operates an HACCP programme and adheres to general rules for good hygiene practice. The animal feed producer is checked and approved/registered by the Norwegian Food Safety Authority.

In addition, national requirements strive to minimise the risk of having *Salmonella* in the animal feed chain and to maintain the present situation of low *Salmonella* contamination [11]. These minimum requirements for prevention and control of *Salmonella* in animal feed are provided for in the national legislation (FOR 2002-11-07 nr.1290: Forskrift om fôrvarer, chapter V and annex 14).

Raw materials, including soybeans, are defined as high risk animal feed material with regard to *Salmonella*. When these raw materials are imported or when by-products of these products are used in the manufacturing of animal feed, the animal feed mill must have a *Salmonella* control program. Importers of animal feed material must ensure that the imported animal feed material is proven free from *Salmonella* contamination according to the fixed sampling programme before it can be received. If *Salmonella* is found in an animal feed mill, it has to be reported to the Norwegian Food Safety Authority, to agree on necessary actions to minimise the risk of having contaminated animal feed on the market; and serotyping of the *Salmonella* must be done.

Because of the regular presence of *Salmonella* in dust from imported soybeans, a dispensation from the requirement of absence of *Salmonella* in imported raw materials is granted to Denofa by the Norwegian Food Safety Authority, provided that evidence of *Salmonella* safe end products is ensured. To comply with this requirement a customized *Salmonella* control programme has been designed for the plant. The programme is approved by the Norwegian Food Safety Authority, and is implemented by the company. Based on the existing control programme, which in principle is the same as that applied in Sweden [12], Swedish Authorities allow soybean meal tested free from *Salmonella* contamination at Denofa to be used in animal feed production in Sweden without additional testing at the time of entry into Sweden, a dispensation from its national requirement.

#### Testing procedures

A control program including sampling at critical control points covers the raw material fed into the process (1), outdoor environment, indicating the potential external *Salmonella* risk to the process (2), and the indoor environment and process equipment, including a special focus on the clean zones and clean process equipment (3). In addition, extensive sampling of the final products is undertaken to verify the absence of *Salmonella* (4). The control program is in accordance with the principles applied for feed mills [5],[6].

Because the different critical control points selected for the sampling varies by plant, they are not presented here. Instead, the testing procedures applied and associated results for isolation of *Salmonella* are presented, with a focus on the contamination status of the incoming and outgoing products plus some overall key data. Sampling from the dust arising during the unloading of the soybean vessels is used for measuring the *Salmonella* contamination of the soybeans entering the plant. During unloading of the vessel representative samples of the accompanying dust are collected and pooled into 24 subsamples, which are analyzed for each vessel.

Before the final product arrives at the meal storage area for the outgoing product, it is automatically sampled from a moving stream every 6 minutes. In each shift sample of 8 hours, representing about 350 tonnes of soybean meal, 80 incremental samples are generated and pooled into one shift sample to be analysed. When products are loaded for final delivery to customers, all of the trucks and meal vessels are sampled again. Samples from all of the vessels are analysed, and 5 trucks per day are randomly chosen for analyses. Samples from all deliveries are kept for reference.

Altogether about 4000 samples are analysed per year in the plant (Table 1). All of these are tested by standard bacteriological procedures [13] according to the NMKL-71 method [14], or by polymerase chain reaction assay (*Salmonella* spp AFNOR NO QUA-18/3-11/02; BAX), which in cases of a positive result are verified with the NMKL-71 method. The analyses of end product samples are done at accredited laboratories. All positive samples are sent to the Norwegian Veterinary Institute for serotyping.

**Table 1:** **Results of** ***Salmonella*** **control at Denofa crushing plant during 2012**

**Sample category**	**Sample**		***Salmonella*** **positive samples**	
	**Number**	**%**	**Number**	**%**
Dust in raw materials (soybeans)	312	8%	36	11.5%
Outdoor environment	61	2%	13	21.3%
Indoor environment/“dirty zone”	228	6%	21	9.2%
Indoor environment/“clean zone”	778	19%	3	0.4%
End product – Soybean meal	2690	66%	0	0%
Total	4069	100%	73	

Efforts to minimize the risk for *Salmonella* contamination of the final product and the build- up of an in house contamination are always applied whenever *Salmonella* is detected. In case of *Salmonella* positive testing in final product samples, or samples from the indoor environment on the end product side (“clean zone”), measures are undertaken, irrespective of serovar, to eliminate the contamination and the risk of contaminated product entering the animal feed chain. The authorities and customers are contacted as needed. All results from the *Salmonella* control activities are reported to the Norwegian Food Safety Authority. The plant is also subjected to regular audits from the same authority.

## Results

**Figure 2:**
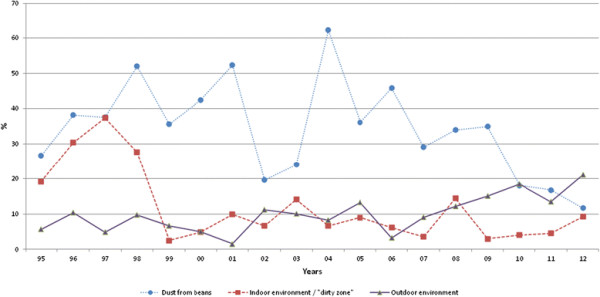
**Isolation of** ***Salmonella*** **from Denofa crushing plant (% of total samples).**

**Figure 3:**
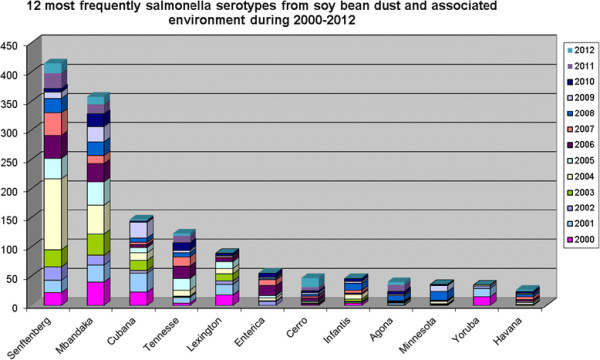
**Serovars of** ***Salmonella*** **isolated from Denofa crushing plant during 2000–2012.**

The results are first presented as an overall isolation of *Salmonella* from soy beans and the internal and external environments of the crushing plant during the whole period 1994–2012 (Figure 2).This is followed by the result of the HACCP based sampling during one year (2012), which is representative of the most recent years (Table 1) and in Table 2 further key data on isolation of *Salmonella* for the period 2000–2012. Finally the isolated serovars of *Salmonella* during the whole period (1994–2012) is presented in Table 3, along with the twelve most common serovars during the period 2000–2012 (Figure 3).

**Table 2:** **Results of** ***Salmonella*** **control at Denofa crushing plant during 2000-2012**

	**Year**													**Mean (M)/Total (T)**
	**2000**	**2001**	**2002**	**2003**	**2004**	**2005**	**2006**	**2007**	**2008**	**2009**	**2010**	**2011**	**2012**	
Total annual number of *Salmonella* positive samples	155	178	81	119	249	147	159	121	144	120	78	79	73	M: 127
Total number of serovars isolated annually	21	26	19	23	20	22	25	23	28	24	16	12	16	M: 21
Indoor environment/“clean zone” - % *Salmonella* positive samples	0	0	0	1	6	11	1	0	2	0	3	1	3	M: 2
Number of stop in delivery of soybean meal to ensure freedom of *Salmonella*	0	0	0	0	0	2	0	0	0	0	0	0	0	T: 2
End product - % *Salmonella* positive samples	0	0	0	0	0	0	0	0	0	0	0	0	0	T: 0

**Table 3:** **Serovars of** ***Salmonella*** **isolated at the Denofa crushing plant per year from 1994 to 2012**

	**1994**	**1995**	**1996**	**1997**	**1998**	**1999**	**2000**
	*Spp.* Agona	*Spp.* Abony	*Spp.* Alabama	*Spp*. Carrau	*Spp*. Indiana	*Spp*. Adelaide	*Spp*. Madelia
	*Spp.* Anatum	*Spp.* Arechavaleta	*Spp.* Gaminare	*Spp*. Derby	*Spp*. Kottbus	*Spp*. Cerro	
	*Spp.* Berta	*Spp.* Braenderup	*Spp.* Give	*Spp*. Glostrup	*Spp*. Minnesota	*Spp*. Eschweiler	
	*Spp.* Bovismorbificans	*Spp.* Brandenburg	*Spp.* Hadar	*Spp*. Lille		*Spp*. Poona	
	*Spp.* Cubana	*Spp.* Sub.species	*Spp.* Infantis	*Spp*. Meleagridis		*Spp*. Rissen	
	*Spp.* Hartford	3.b. (S. Diarizonae)	*Spp.* Kentucky	*Spp*. Miami		*Spp*. Yoruba	
	*Spp.* Lexington	*Spp.* Enteritidis	*Spp.* Kingston	*Spp.* Ohio			
	*Spp.* Llandoff	*Spp.* Sub.species	*Spp.* Muenster	*Spp*. Ouakum			
	*Spp.* Mbandaka	1 G.O.:7	*Spp.* Pakistan	*Spp*. Redeney			
	*Spp.* Montevideo	*Spp.* Havana	*Spp.* Tennesse	*Spp*. Saintpaul			
	*Spp.* Newport	*Spp.* Houtenae 51:a					
	*Spp.* Oranienburg	*Spp.* Lagos					
	*Spp.*ParatyphieB.	*Spp.* Lansing					
	var.-Java/ Abony	*Spp.* London					
	*Spp.* Senftenberg	*Spp.* Manhatten					
	*Spp.* Thompsen	*Spp.* Muencen					
	*Spp.* Typhimurium	*Spp.* Panama					
	*Spp.* Worthington	*Spp.* Paratyphie B. Fagt. Dundee					
**Total**	17	16	10	10	3	6	1
	**2001**	**2002**	**2003**	**2004**	**2005**	**2006**	**2007**
	*Spp*. Schwarzengrund	*Spp*. Abaetetuba	*Spp*. Bere	*Spp*. Langeveld	*Spp*. Dallgow	*Spp* Javiana	*Spp*. Corvallis
	*Spp*. Sundsvall	*Spp*. Enterica	*Spp*. Heidelberg	*Spp*. Sandiego			*Spp*. Soerenga
		*Spp*. Goldcoast		*Spp*. Saphra			
		*Spp*. Morehead					
**Total**	2	4	2	3	1	1	2
	**2008**	**2009**	**2010**	**2011**	**2012**	**Total 1994- 2012**	
	*Spp*. Benefica	*Spp*. Gatow	*Spp*. Bareilly	*Spp*. Aarhus	*Spp*. Chester		
	*Spp*. Breda	*Spp*. Maricopa	*Spp*. Gloucester	*Spp*. Salamae	*Spp*. Kedougou		
	*Spp*. Ealing	*Spp*. Regent	*Spp*. Thomson	*Spp*. Winston			
	*Spp*. Ruiri						
	*Spp*. Vejle						
**Total**	5	3	3	3	2	94	

### Contamination rate

As can be seen from Figure 2, *Salmonella* are frequently isolated from the soy bean dust upon importation. During the period studied, a total of 6048 samples taken from the soy bean dust were analyzed out of which 2074 (34.3%) were contaminated by *Salmonella*. All positive samples are serotyped. Dust following all imported shiploads of soy beans from South America was found to be *Salmonella* contaminated, and was more contaminated than dust from beans coming from other origins (Canada and eastern Europe). The mean annual proportion of detected *Salmonella* contaminated samples from all imported shiploads was 34.3% with a range from 11.5 to 62.3%. During the period 2010–2012 the contamination rate decreased to the lowest level recorded (11.5%), for an unknown reason. During 1994–1998 the average contamination rate in the indoor environment of the “dirty zone” was close to 30% (29.1), but after substituting old equipment in 1998 the contamination dropped to a rather stable level at an average level of 7.8% throughout the remaining period (1999–2012) examined. The contamination of the outdoor environment was at a similar level (6.8%) up to 2006, when for an unknown reason a slight increase in positive *Salmonella* findings was recorded, with an average contamination rate of 17.6% during 2007–2012. Due to the fact that the indoor contamination in the “clean zone” was very low, as seen in Table 1 and Table 2, and that no *Salmonella* contamination was recorded in the end product during the studied period, those data are not included in Figure 2 because they should largely follow the baseline (0%).

The overall data for the sampling in different sections of the plant during one year (2012), representative for the most recent years of the study, is presented in Table 1. In addition to data presented in Figure 2, it can be seen that the indoor environmental contamination in the “clean zone” was very low (0.4%). It can also be seen that *Salmonella* was not detected in any of the 2690 samples tested from the final product, the soybean meal. This was also the result for all of the previous years of the period studied for the final meal delivered to the customers/animal feed mills (Table 2). In total >4000 samples were tested annually from the plant, out of which 85% were from the “clean zone”.

The total annual number of *Salmonella* positive samples from the plant from 2000–2012 is presented in Table 2. This varies from 73 to 249, and mainly reflects contamination of dust from soy beans. For example, during 2004 when that contamination was >60% (Figure 2), 249 samples were positive for *Salmonella*. This high contamination rate is most likely also the reason for the increased isolation of *Salmonella* on “the clean side” during that and the subsequent year, 6 and 11 isolations respectively, and the two stops in delivery of soybean meal during 2005 to ensure freedom of *Salmonella* (Table 2).

### Isolated serovars

During 1994–2012 94 different serovars of *Salmonella* were isolated from the samples examined (Table 3). Annually (2000–2012) on average 21 different serovars were isolated, with a range of 12 to 28 (Table 2). Since the program was started in 1994, serovars not previously detected were listed during all years. Some of serovars occurred more frequently, which is illustrated in Figure 3. Five serovars dominated: *S.* Seftenberg, *S.* Mbandaka, *S.* Cubana, *S.* Tennesse and *S.* Lexington. The remaining serovars were isolated less frequently and less regularly.

## Discussion

This paper shows that soy beans imported to a Norwegian crushing plant frequently were contaminated by a wide range of different serovars of *Salmonella*. During the 19 years studied (1994–2012) all imported shiploads from South America were *Salmonella* contaminated. The mean proportion of *Salmonella* positive dust samples was around 34% with a range from 12 to 62%. In spite of a continuous exposure of *Salmonella*, the plant through a HACCP based program and associated control measures could produce soybean meal intended as animal feed material without any signs of *Salmonella* contamination. This is to our knowledge the first study that demonstrates that this can be done under commercial and industrial conditions. Earlier this was also reported from a Swedish rape seed crushing plant [9].

It is difficult to find data on *Salmonella* contamination in soy beans intended for animal feed. However, the frequent isolation of *Salmonella* from the soy beans in this study is rather similar to the *Salmonella* contamination of soybean meal and other vegetable protein produced in crushing plants, as reported from different countries [15]. In a recent comprehensive study based on an annual examination of up to 80,000 lots of animal feed in Poland, 15.0% and 15.4% of imported lots of soy and rape seed meal respectively were found to be *Salmonella* contaminated [16]. The corresponding data for products produced within Poland were 6.3% and 7.7%. In Sweden 14.6% of 795 imported consignments of soybean meal were found to be contaminated by *Salmonella* during 2004–2005, and when considering only imports (mostly from South America) the level was approximately doubled [6],[17], and this higher level has regularly been found in the Swedish animal feed control where all consignments of soybean meal are tested before introduction to the animal feed mills [18]. However a direct comparison of data from different studies is difficult due to differences in sampling and testing procedures [19]. This is illustrated in a Danish study where a low sensitivity sampling program (i.e. one sample per batch/shipment of imported soybean meal) detected 35 isolates of *Salmonella* during 1994–2003 compared to 1086 isolates when 22 shipments were investigated during 2004 with a more intensified sampling [20].

Due to the fact that the production process and the associated risk for *Salmonella* contamination is rather similar in crushing plants and animal feed mills, the experiences from the control of *Salmonella* in in animal feed mills can be applied to the crushing plants [15]. This means that the sources of the frequently occurring *Salmonella* contamination of the soybean meal (as referred to above), in addition to being a contamination of the soy beans that might not have been eliminated in the crushing process, more likely is a recontamination from an in house contamination of the crushing plant, initially introduced by contaminated soy beans. As indicated from this study, the soy beans can therefore be considered to be a risk product for *Salmonella* contamination of the crushing plants. Based on the experiences from animal feed mills, an effective HACCP based control for the early detection and prevention of the buildup of an in house *Salmonella* contamination should therefore also be a way to avoid recontamination of the end product in crushing plants, subsequent to the crushing process that should readily eliminate contaminating *Salmonella* bacteria [15],[21],[22].

The effectiveness of the HACCP based control in this study is indicated by the low prevalence of *Salmonella* contamination in the “clean zone” of the plant (Table 2), which is a prerequisite for the successful prevention of *Salmonella* in the end product. When *Salmonella* contamination occurs in in the “clean zone”, sampling is increased and measures are taken to eliminate the contamination. On a very few occasions, in fact only twice since 2000, the delivery of meal to customers was stopped to ensure that no *Salmonella* contaminated meal was delivered. These events occurred during 2005, following a period of heavy *Salmonella* contamination of incoming soy beans – up to 62% (Figure 2). Successful prevention can thus not be ensured only by testing the end product [2],[15]. In addition to the fact that such approach would require substantial sampling to overcome problems in detecting a low concentration and uneven distribution of *Salmonella* contamination, it would also be too late during industrial conditions. Long term experiences from Sweden have also showed that soybean meal from Denofa is a *Salmonella* safe animal feed material, with no known contamination of animal feed mills and no subsequent spread to poultry and swine, as sometimes has been observed for soybean meal from other sources [6],[17].

The *Salmonella* contamination of the soy beans during the study period involved 94 serovars, and annually approximately 21 (12–28) different serovars were isolated. Five serovars dominated (*S.* Seftenberg, *S.* Mbandaka, *S.* Cubana, *S.* Tennesse and *S.* Lexington). The remaining serovars were isolated less frequently and less regularly. Also other studies have demonstrated that animal feed or animal feed ingredients often are contaminated by a wide range of serovars of *Salmonella*. In Sweden e.g. 38 serovars were isolated from animal feed-associated sources during a 2-year period [6], and in the Danish study referred above 50 different serovars were isolated during 1994–2003 [20].

It is sometimes stated that those serovars of *Salmonella* contaminating animal feed seldom cause disease in humans [1]. It is therefore interesting to note that the serovars isolated from the plant included 9 of the 10 (90%) most common serovars isolated from clinical cases of salmonellosis in humans in the EU during 2012 [23]. In addition they also included well known animal pathogens like *Spp*. Typhimurium and Enteritidis which both also belong to the top ten serovars of human isolates. *Sp*. Enteritidis has earlier also demonstrated its potential for a pandemic spread in both poultry and to humans [24]. A similar result was previously found in Sweden where four (10.5%) of the 38 animal feed associated serovars were among the 10 most frequent isolates from human cases of salmonellosis in the EU [6].

In the absence of animal derived proteins, which since 2001 largely have been banned as animal feed ingredients to prevent the spread of BSE, vegetable protein can be considered to be the major risk animal feed material for introducing *Salmonella* into the animal feed chain and animal farms [2]. As long as no information seems to be available on why and where the *Salmonella* contamination of soy beans occurs, and how to prevent that contamination, the best way to minimize the risk for spread of the pathogen further in the animal feed and food chain would be to eliminate the contamination already in the crushing plant, as shown in this study. This would be in line with the recommendations by the FDA in 1991 and by Crump *et al.*[1] that a *Salmonella*-negative standard for animal feed should be implemented.

## Conclusions

The elimination of *Salmonella* contamination of vegetable animal feed materials like soybean meal already in the crushing plants producing the meal, can be suggested as an effective way to minimize the risk of *Salmonella* exposure of the animal feed mills and contamination of the subsequent animal feed chain. Such an approach is considered to be realistic, since the production process in the crushing plants normally reaches temperatures that should kill contaminating *Salmonella* bacteria.

## Competing interests

The authors declare that they have no competing interests.

## Authors’ contributions

MW is the main author of the manuscript, and transferred and analysed the original data to its current form in close cooperation with TK. TK is Quality Director and responsible for all quality issues, incl. *Salmonella*, the import and production chain of soy beans to soy meal during the whole study period and co-writer of the associated text and assessments. Both authors read and approved the final manuscript.
